# Glycosphingolipid expression in squamous cell carcinoma of the upper aerodigestive tract

**DOI:** 10.1016/S1808-8694(15)30029-X

**Published:** 2015-10-19

**Authors:** Marcilio Ferreira Marques Filho, Fernando Walder, Helio K. Takahashi, Luciana L. Guimarães, Ameria K. Tanaka, Onivaldo Cervantes, Anita H. Straus

**Affiliations:** a PhD in Sciences – Federal University of São Paulo, Former post-graduate student from the Otolaryngology and Head and Neck Surgery Department of the Federal University of São Paulo. Physician from the Department of Otolaryngology and Head and Neck Surgery at the Santa Casa de Misericórdia de Itabuna Hospital; b PhD in Medicine – Medical School – University of São Paulo, Assistant physician – Head and Neck Department – Federal University of São Paulo.; c Full Professor of Molecular Biology – Biochemistry Department – Federal University of São Paulo.; d Post-Graduation Student in Molecular Biology – Department of Biochemistry – Federal University of São Paulo.; e Post-Graduation Student in Molecular Biology – Department of Biochemistry – Federal University of São Paulo.; f Associate Professor of Medicine – Federal University of São Paulo, Chief of the Head and Neck Department – Federal University of São Paulo.; g Associate Professor – Federal University of São Paulo. Head and Neck Department and Molecular Biology Department (Glycoconjugated Immunochemistry Sector) – Federal University of São Paulo, Paulista School of Medicine (UNIFESP-EPM)

**Keywords:** glycosphingolipids, squamous cell carcinoma, gangliosides, head and neck neoplasms

## Abstract

Glycosphingolipids are integral constituents of cellular membrane, arranged in rafts, and with neoplasic cell antisocial behavior, like uncontrolled cell growth, invasiveness, and metastatic potential.

**Aim:**

However, there are few studies about glycosphingolipids (GSL) expression in squamous cell carcinoma (SCC). Since GSL are known to be tumor-associated markers we decided to perform a prospective study on the GSL profiles of SCC.

**Method:**

Specimens of 33 SCC and normal mucosa were obtained and GSLs were extracted and purified by reverse-phase chromatography on C18 column and alkaline hydrolysis in methanol. GSLs were quantified using densitometry of orcinol-stained HPTLC plates.

**Result:**

A significant increase of GSLs in SCC (3.57µg/mg) was observed as compared to normal mucosa (1.92µg/mg). In SCC, an increase of 2 to 3 times in the amounts of CDH, CTH, Globoside, and GM3 was observed in comparison to normal mucosa. The identification of GM3 as well as its increased expression in SCC was confirmed unequivocally by HPTLC immunostaining and indirect immunofluorescence using MAb DH2 (anti-GM3). BY analyzing SCC and normal mucosa CMHs by GC/MS, normal mucosa expresses only glucosylceramide whereas SCC cells express both glucosylceramide and galactosylceramide.

**Conclusion:**

The increase in the amount of GSLs in tumor tissue may represent changes of cell membrane microdomains resulting from the malignant transformation process, which is responsible for greater cell-cell or cell-matrix interaction thereby increasing their potential for infiltration and metastasis.

## INTRODUCTION

Besides outlining cell limits, the cell membrane has other important functions. It is responsible for maintaining ion gradients, the communication with other cells and with the external medium through transmembrane receptors, located in specialized areas called microdomains^2^. Alterations in microdomains and their components, GSLs among them, are related to malignant transformation processes, a much studied theme, especially in melanomas, breast, colon and lung cancers^1^.

The cell membrane is made up of a double lipid, and a protein layer, perpendicularly arranged, totally or partially soaked within both lipid layers^2^, ^3^, ^4^. Most cell membrane lipids are phospholipids. Besides phospholipids, cholesterol and glycolipid molecules are also present in large amounts^2^, ^5^ in the cell membrane. Glycosphingolipids (GSL) are the most abundant glycolipids in the cell membrane, being formed by a ceramide molecule (sphingosine linked to a fatty acid) with one or more sugar residues linked to its primary carbon^5^, usually present in specialized areas known as microdomains^2^. These microdomains, or lipid rafts, are cell membrane areas, formed by GSL, cholesterol and proteins, which work as a platform where some proteins attach to, allowing them to act together and be transported within the double lipid layer^6^, ^7^. Important cell functions are attributed to microdomains, such as cell signaling modulators, cell adhesion mediators and also antigenic function^8^.

Hakomori states that the GSLs, the gangliosid among them (GSLs with syalic acid residues), have their expression increased in tumors due to alterations in their glicosylation, observed along his 35 years of research, in vitro and in vivo changes in GSL glisosilation^1^, ^9^.

It is well shown the aberrant glicosylation in melanomas through dissyaloganglioside (GD3) expression, gangliosid expressed in melanomas and not in the melanocyte. This ganglioside is related to the fast growth and especially to the undifferentiated and amelanocytic melanoma form, working as a tumor marker^10^, ^11^. Colon, breast and lung malignant tumors also have GSLs as tumoral markers, and there is pre-clinical research about it^12^, ^13^. For melanoma, for which research is more advanced, we already have vaccines produced with anti-GSL monoclonal antibody (MAb), approved for clinical use in Canada and Australia^14^.

However, as far as head and neck SCC (squamous cell carcinoma), especially in upper airways and digestive tracts, there are few papers showing GSL expression in the SCC differentiation or malignant transformation, despite the fact that one of the first antibodies developed against GSL be able to identify SCC LacCer^1^, ^15^. Only Bolot et al. (1998, 1999) assessed GSL expression in head and neck carcinoma and normal tissue cells, notwithstanding these papers leave doubts in regards of histologic types and the anatomical sites of the tumors studied.

Since the SCC GSL expression is not very much studied, we decided to carry out a prospective study aiming at assessing SCC GSL expression in the upper airway and digestive tract.

## METHODS

We carried out a prospective study in 33 upper airway and digestive tract SCC samples and normal cells of patients operated at the Head and Neck Department - UNIFESP-EPM, of those 15 were larynx tumors, 9 were oropharynx, 3 hypopharynx and 6 oral cavity tumors. The patients were not separated as to age and gender.

The collected material was processed in the glycosphingolipids lab of the glycoconjugated Immunochemistry Sector – Biology Molecular Department – Federal University of São Paulo – Paulista School of Medicine.

The material was homogenized and the GSL was extracted with the use of IPA/hexane/ H20 (55:20:25 v/v/v) twice and once with chloroform/methanol (2:1 v/v).

After extraction, the GSL was purified by reverse hydrolysis chromatography in C-18 column and methanol alkaline hydrolisis^16^, ^17^. GSL quantification was carried out by HPTLC plaque densitometry, dyed with orcinol using Shimadzu CS 9000 densitometer at 525nm, having GM3 (Sigma®) as standard reference in the concentration of 0,1µg^17^, ^18^. In order to better visualize the gangliosides, the plaques were dyed with resorcinol. The gangliosides were seen as purple spots^19^.

We analyzed the reactivity of GSL expressed for MAb DH2 (anti-GM3) by immune coloring of the HPTLC plaques and indirect immune fluorescence.

With the GSL identified and quantified, we carried out the statistical study through the t-independent test, and (ANOVA) variance analysis with a 5% significance level.

In order to identify sugar residues present in the normal mucosa and SCC CMH, the material was individualized by preparative HPTLC and the CMH corresponding fraction was derived (metanolysis, re-N-acetilation and trimetylsylation) and analyzed in gaseous CP-3800 chromatographs coupled to Quadrupole MS 1200L Varian® (GC/MS) mass spectrometer.

The Ethics and Research Committee of the Federal University of São Paulo approved the study.

## RESULTS

**Figure 1:**
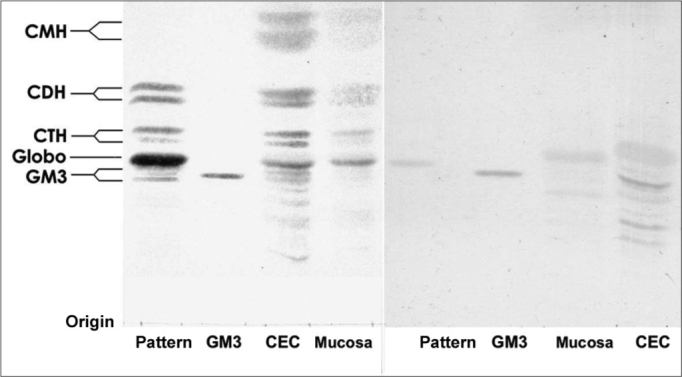
Orcinol/H2SO4 dyed HPTLC slide. (Left) and (right) resorcin dyed HPTLC - Pattern – erythrocyte GSL pattern. GM3 – GM3 Pattern (Sigma®). SCC – SCC GSL. Normal mucosa normal – normal mucosa GSL.

Analyzing the data obtained we see that there was no difference in the GSL types expressed in SCC when we compare it to the normal mucosa, the main GSL expressed were GM3, globoside, CTH, CDH and CMH. However, we saw an increase in GSL expression in SCC, and thus it is possible to have an idea about this difference analyzing the intensity of SCC GSL band coloring in relation to the normal mucosa (Figure 1).

We saw a significant increase in SCC GSL when compared to the normal mucosa. For this comparison we used the t-independent test, where p>0.001 (Table 1).

**Graph 1:**
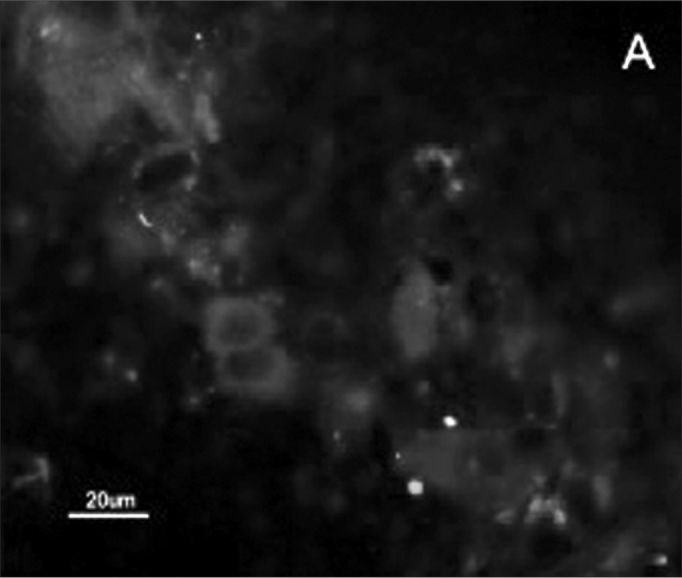
Average of upper airway and digestive tract and SCC GSL quantity.

In order to determine which GSL are found significantly increased in SCC, we used the ANOVA test, and its results proved to be significant for all effects. Through multiple comparisons we identified a greater expression of all GSL in SCC when compared to the normal mucosa, except for CMH (Graph 1).

Other gangliosides such as GM1 and GM2 were also expressed in some SCC samples, in smaller amounts.

**Figure 2:**
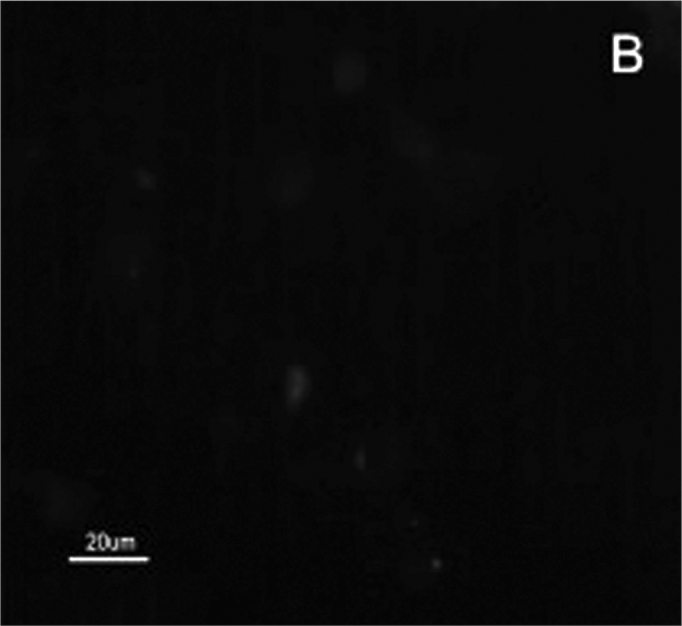
Indirect immune fluorescence microphotography where we see the difference in SCC cell reactivity in comparison to normal mucosal cells with MoAb DH2 (AntiGM3). - **A.** DH2 immune dyed SCC cells (green). **B.** DH2 marked normal mucosa cells (green). **C.** DAPI dyed SCC cell nuclei (blue). **D.** DAPI dyed normal mucosa cell nucleus (blue).

The SCC and upper airway and digestive tract cells reactiveness to MAb DH2 (anti-GM3) is shown by imprint indirect immune fluorescence and by immune coloring of HPTLC plaques. We noticed the important difference in SCC expressed GM3 reactiveness when compared to that of the upper airway and digestive tract normal mucosa in both indirect immune fluorescence (Figures 2) and immune coloring. There was no normal mucosa or SCC cells reactivity to MAb MEST-1 (anti β-galactofuronose), used as inespecific antibody.

**Figure 3:**
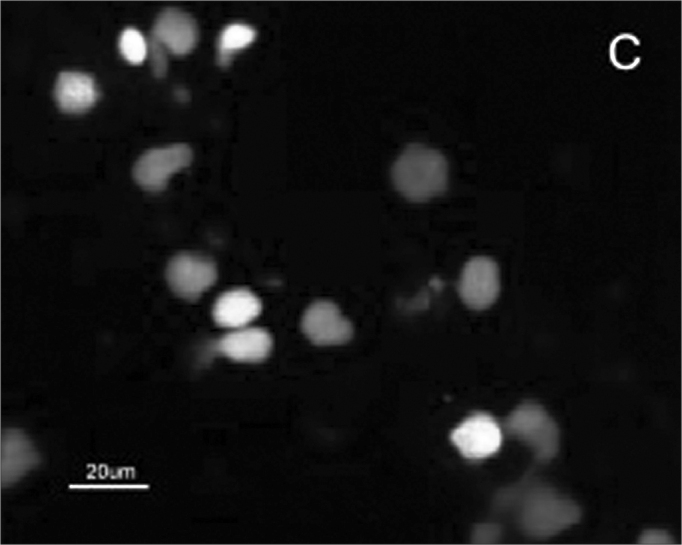
CMH molecules mass spectrometry. The upper lines (A and B) show sugar residues identification in upper airway and digestive tract mucosa CMH fraction. Lines C and D correspond to the identification of sugar residues in SCC CMH fraction. Gal - galactose Glc - glucose.

Sugar residues which are part of the CMH were analyzed by GC/MS, comparing the retention time of glucose and galactose patterns with the retention times obtained from CMH and SCC and that of the normal mucosa. Through GC/MS we detected the presence of two peaks for each type of sugar, corresponding to the α and β isomers of each molecule, with retention times of 31.325 and 31.977 for galactose (Gal), and 32.527 and 32.894 for glucose (Glc). We noticed both SCC glucosylceramide (GlcCer) and galactosylceride (GalCer) expressed, whilst the upper airway and digestive tract mucosa express glucosylceramide only. SCC GalCer represents 35% of the CMH fractions (Figure 3).

## DISCUSSION

Alterations in both GSL structure and expression in cell neoplastic growth, differentiation and transformation is a well documented fact in the literature, being the goal of research for decades now^9^, ^20^. However, as far as head and neck, and mainly upper airway and digestive tract SCC are concerned, there are just a few papers showing GSL expression in the SCC differentiation or malignant transformation process.

The only two articles, of the same group, that assessed GSL expression in carcinoma cells compared to head and neck normal tissue, leave doubts as to the histological type and the anatomic sites of the tumors studied; besides, the small number of tumors studied by this group, six cases only, do not correctly represent SCC GSL expression^21^, ^22^. Another description of SCC GSL expression profile was carried out when they studied GSL expression during the differentiation process in SCC cells in the retromolar region^20^.

**Figure 5:**
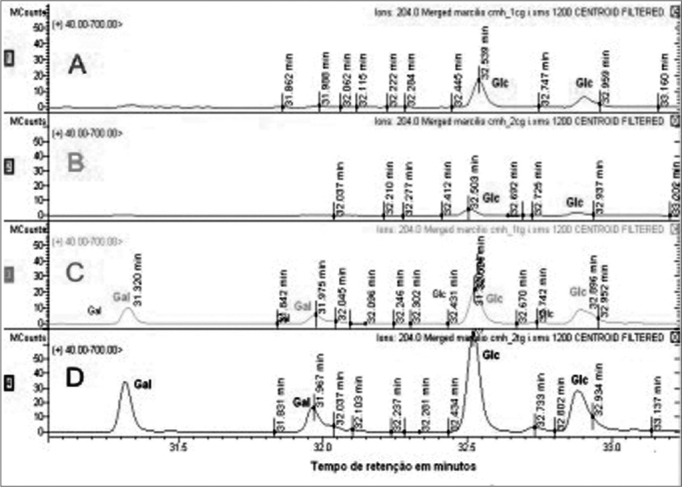
GSL quantity average in upper airway and digestive tract mucosa and SCC.

**Figure 6:**
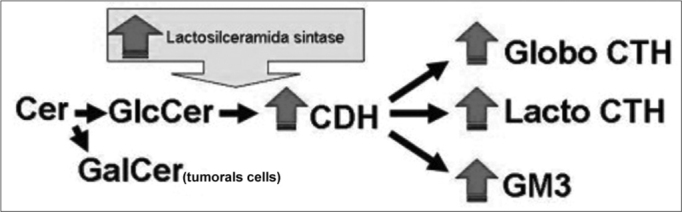
GSL quantity average in upper airway and digestive tract mucosa and SCC.

**Figure 7:**
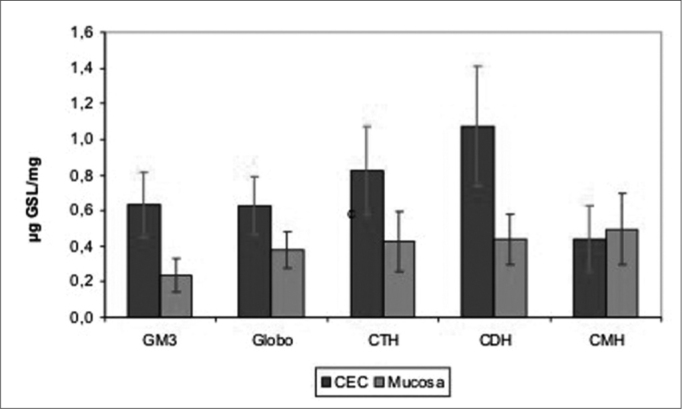
GSL quantity average in upper airway and digestive tract mucosa and SCC.

**Figure 4:**
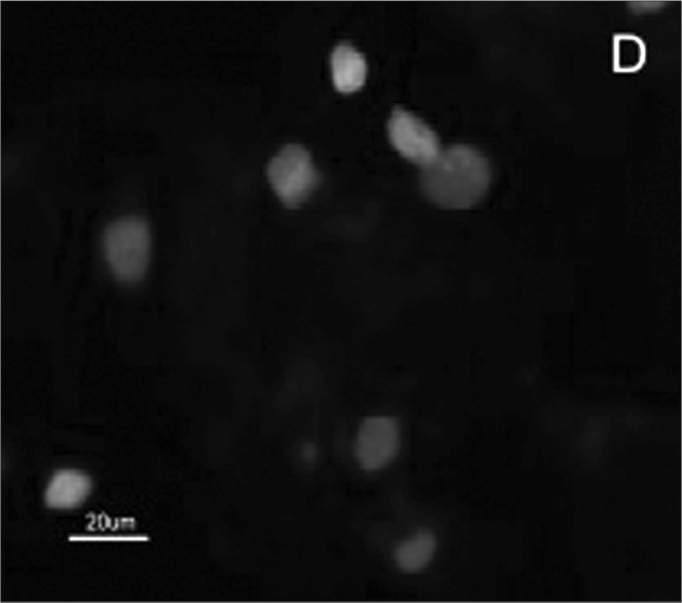
Proposed diagram for the GSL synthesis pathway to explain the increase in these molecules expression in upper airway and digestive tract and SCC. The greater lactosylceramide sinthasis activity would increase GM3, CTH and CDH expression and, consequently would reduce Glc supply for GlcCer synthesis, thus the SCC cells need to use Gal in order to produce GalCer and keep the cell membrane structure.

Our study showed a significant increase in SCC total GSL in the upper airway and digestive tract in relation to the normal mucosa, especially due an increase in GM3, globoside, CTH and CHD expressions. CMH is the only exception, because we detected similar quantities of it in both SCC and normal mucosa. Analyzing the aforementioned results and the ganglioside synthesis metabolic pathway, we may suppose that UDP-Gal: Glucosilceramidaβ1-4galactosiltrasnferase (lactosylceramide sinthasis) activity, responsible for the addition of a galactose residue to GlcCer, turning it into CDH, is increased due to the neoplastic transformation process. Contributing to this hypothesis is the fact of it having been found in the GalCer tumoral CMH fraction, suggesting that the GalCer would be synthesized for GlcCer replacement, thus maintaining the cell membrane structure (Figure 4).

The increase in total GSL and the presence of CMH as GalCer was also seen by Bolot et al. (1998)^22^.

Confronting GSL expression seen in our work with literature data, the only difference found was the presence of GD3, seen by Tatsumura et al. (1988) and Bolot et al. (1998)^20^, ^22^. And, as we analyze GSL expression in head and neck SCC fragment collection obtained from six patients, Bolot et al. (1999) found NAc-GM1 only in SCC, in minimal amounts^21^.

We attribute this ganglioside expression difference we saw in comparison to those described by Bolot et al. (1998 and 1999) and Tatsumura et al. (1988), to two aspects^20^, ^21^, ^22^:

1. For believing it to be more representative, we decided to identify the GSL in each harvested tissue fragment and analyzed them individually, as Bolot et al. (1998 and 1999) did, or according to Tatsumura et al. (1988) who employed SCC cell strains^20^, ^21^, ^22^.
2. There may really be differences in GSL expression, once the literature shows, quite well, the large differences existing as to SCC genotype and phenotype, fact that has made it impossible to, so far, establish reliable and clinically useful conclusions^23^, ^24^, ^25^.

Bolot et al. (1998 and 1999) paper calls our attention to the quantity of GD3 similar to GM3, and this reinforces the hypothesis of a distinct pattern in GSL expression in the tumors studied by these authors, in relation to the samples analyzed in our study^21^, ^22^.

Tatsumura et al. (1988) observed in SCC cell lineage (SqCC/Y1) cultivated in a complete culture medium, a reduced GD3 expression. Only by cultivating SqCC/Y1 cell lines in a culture medium without serum was possible to detect a significant increase in GD3 expression.

It is curious to see GM3 increase in tumoral tissue, because its increase is related to the inhibition of the cell growth process. GM 3 inhibits epithelial growth factor receptors self phosphorilation^26^, ^27^, and may induce apoptosis via Bcl-29. Another hypothesis, contrary to the previous explanation, is the possibility of GM3 act as a second messenger in the growth process regulation; however, we still need more detailed studies to prove the role of GM3 in cell growth^27^.

The main possibility of using GSL to aid in the diagnosis and treatment of malignant neoplasia is based on structure alteration and/or GSL conformation, more precisely that of the carbohydrates presented in these molecules, thus allowing MAb production, that specifically recognizes tumor-related GSL.

Because we had GM3 expression in both the mucosa and in the SCC in this study, it would be expected a MAb DH2 reactivity proportional to the concentration of these GSLs in both tissues, however this was not observed (Figures 2). We believe that the difference in GM3 reactivity is justified by its conformational placement in the microdomains when expressed in greater quantity. We know, for instance, that the MAb M2590 (anti-GM3) only detects GM3 in the cells where there is relevant GSL expression, and there is the trend of this glycoconjugated in grouping itself in a way that is different in microdomains when there is any increase in its expression, thus, increasing MAb M2590 reactiveness. These results suggest the need of a critical concentration in order to achieve an optimum interaction with MAb DH2.28

As mentioned before, there is an increase in GM3 expression in cell growth, and experimentally we have observed a DH2 (anti-GM3) inhibitory effect in melanoma cell growth in vivo and in vitro^29^. On the other hand, we saw that as we add GM3 to our culture medium, we have a cell growth inhibition. These results have contributed to the development of clinical and experimental research trying to employ GSLs and mAbs (anti-GSL) in the diagnosis and treatment of malignant neoplasias1. As a result, we introduced mAbs specially guided against GSL and associated to different tumors. These tumor cells associated GSL guided mAbs have been used in cytological and pathology exams trying to differentiate benign lesions from malignant tumors^17^. Despite promising results, so far, the use of GSLs in cancer diagnosis and treatment is still restrict to some types of tumors, such as melanomas^9^, ^30^, ^31^.

The use of GSL as anticancer vaccines antigen^2^ is a possibility that has aroused the interest of the scientific community in current days. In a phase III study, in which we employed lung cancer associated antigens, we obtained a survival of 70% compared to 49% for stages I and II in 5 years^32^. The largest number of anticancer vaccines were developed and tested against melanoma cells, using mAbs anti-GM3, GM2 and GD3. Other vaccines have been produced against tumors such as, for instance, breast and intestine, however, they remain as clinical assays.

Only in Canada and Australia there are clinically approved anticancer vaccines for melanoma, where metastasis patients had a survival increase of 25% to 50% in five years. Despite these promising results, most of the anticancer vaccines developed so far remain as research projects, not being approved by the north-American (FDA) and European regulating agencies for clinical use^14^.

Therefore, the possibilities of using GSL and MAb that recognize these GSL to help in the diagnosis and treatment of SCC is promising, as is the case with the melanoma, however, we need further SCC GSL structural and immunochemical research, as well as for the many types of head and neck tumors.

## CONCLUSION

The results from this research work allow us to conclude that there is a significant GSL expression increase in upper airway and digestive tract SCC expression when compared to the normal mucosa of this region. This increase in expression happens especially in GM3, globoside, CTH, CDH; and the main hypothesis for this greater expression in malignant transformation processes would be related to an increase in Lactosylceramide synthase quantity and activity.
